# Supplementary material for: The microbiome biomarkers of pregnant women’s vaginal area predict preterm prelabor rupture in Western China
Source: Front Cell Infect Microbiol. 2024 Oct 31;14:1471027. doi: 10.3389/fcimb.2024.1471027 (PMC11560878; doi:10.3389/fcimb.2024.1471027)
Supplement: Supplementary file 1 [file DataSheet1.zip › compare_1/Community/Structure_Barplot/order/order.taxon.Barplot.Legend.With.superkingdom.pdf]

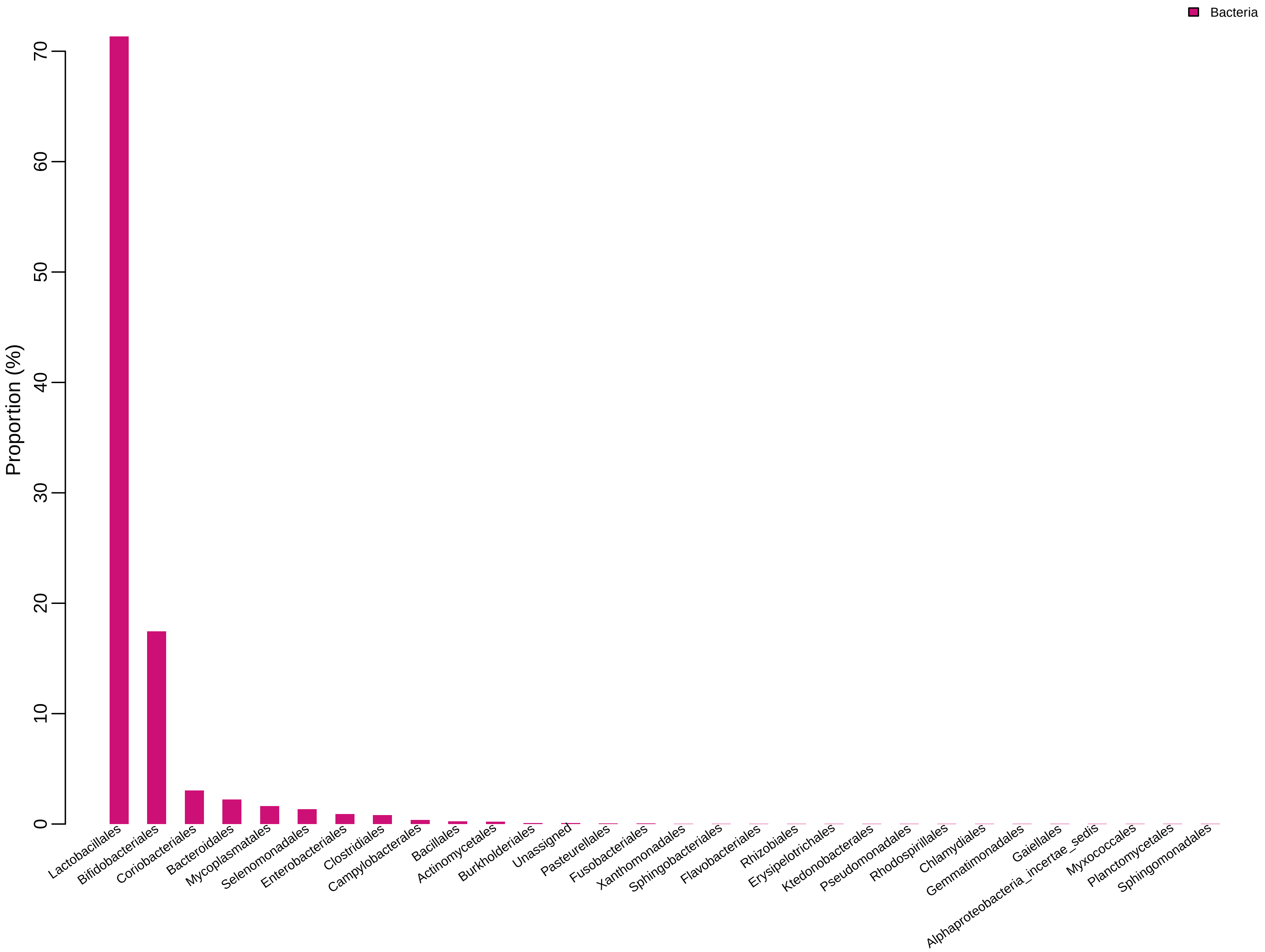

Bacteria
Proportion (%)
0
10
20
30
40
50
60
70
Lactobacillales
Bifidobacteriales
Coriobacteriales
Bacteroidales
Mycoplasmatales
Selenomonadales
Enterobacteriales
Clostridiales
Campylobacterales
Bacillales
Actinomycetales
Burkholderiales
Unassigned
Pasteurellales
Fusobacteriales
Xanthomonadales
Sphingobacteriales
Flavobacteriales
Rhizobiales
Erysipelotrichales
Ktedonobacterales
Pseudomonadales
Rhodospirillales
Chlamydiales
Gemmatimonadales
Gaiellales
Alphaproteobacteria_incertae_sedis
Myxococcales
Planctomycetales
Sphingomonadales
